# Natural products‐induced cancer cell paraptosis

**DOI:** 10.1002/fsn3.4461

**Published:** 2024-09-12

**Authors:** Haitham Al‐Madhagi

**Affiliations:** ^1^ Biochemical Technology Program Dhamar University Dhamar Yemen

**Keywords:** cancer, natural products, paraptosis, programmed cell death

## Abstract

Cancer cell can be killed in a programmed way by natural products in a process known as paraptosis.

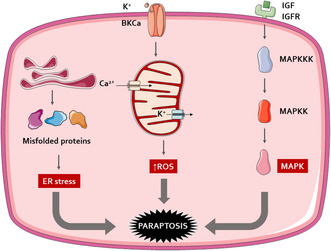

## 1 INTRODUCTION

Most current cancer treatments work by triggering cell death processes, such as apoptosis, autophagy, and paraptosis to halt tumor growth. These treatments, including radiation and chemotherapy, primarily cause cancer cells to die through apoptosis. While tumors often reduce in size initially, they tend to return within 6–18 months, more aggressive and resistant to the same treatments. This recurrence happens as the tumors develop ways to avoid apoptosis (Wepsic & Hoa, 2023). Moreover, even though these treatments release a significant amount of tumor‐specific substances, such as antigens, they fail to create a durable immune response or tolerance against the tumor. On the flip side, triggering programmed (nonapoptotic) cell death in cancer cells is regarded as a promising anticancer therapeutic strategy (Zhou et al., 2018).

## 2 PARAPTOSIS

Paraptosis is a type of programmed cell death (PCD) that is morphologically and biochemically distinct from apoptosis and necrosis. Paraptosis is characterized by cytoplasmic vacuolation, independent of caspase activation and inhibition, and lack of apoptotic features such as membrane blebbing, chromatin condensation, and nuclear fragmentation. Paraptosis can be triggered by various stimuli, such as growth factors, natural compounds, metallic complexes, and photodynamic therapy (Xu et al., 2024). Paraptosis has been implicated in various physiological and pathological processes, such as development, neurodegeneration, viral and bacterial infection, and cancer. Additionally, it has been shown to induce antitumor effects in cancer cells by modulating the tumor microenvironment, the immune system, and the signaling pathways involved in cell survival and proliferation. Indeed, paraptosis is a relatively new and emerging field of research, and there are many challenges and opportunities for further exploration and understanding of this unique cell death mode (Hanson et al., 2023).

Paraptosis is a unique type of PCD that differs morphologically from apoptosis and necrosis, defined by cytoplasmic vacuolation due to dilation of the endoplasmic reticulum (ER) and mitochondria, lack of nuclear fragmentation, chromatin condensation, and apoptotic body formation, rounded cells with cytoplasmic reorganization visible under light microscopy, physical enlargement and swelling of mitochondria and ER attributed to intracellular ion imbalance and osmotic lysis, and rupture of swollen organelles releasing “danger signals” such as HMGB1, heat shock proteins, and proteases, leading to inflammation (Lee et al., 2016; Yan et al., 2020). Unlike apoptosis, paraptosis lacks the hallmark features of membrane blebbing, chromatin condensation, and nuclear fragmentation, with apoptotic cells undergoing shrinkage and forming apoptotic bodies while paraptotic cells show cytoplasmic vacuolation and rounding up. In contrast to necrosis, paraptosis is a PCD involving gene expression, not just a result of injury, with necrosis causing lysis of the cell membrane while paraptosis maintains membrane integrity until late stages; paraptosis shares some similarities with type III PCD, previously described as “cytoplasmic” or “type 3 cell death,” and the morphological changes resemble those seen during development of the nervous system (Kessel, 2019, 2020).

## 3 MECHANISMS OF PARAPTOSIS

**FIGURE 1:**
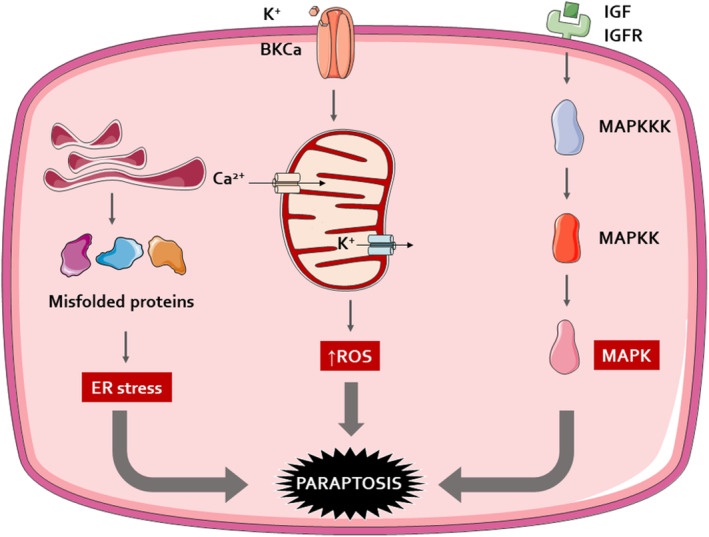
Proposed mechanism of paraptosis activation. Paraptosis was initially discovered to be instigated by IGFIR and stimulate the MAPK signaling pathway. Next, it has been evidenced to be linked to other factors, including TrxR1 inhibition, GSH depletion, intracellular Ca^2+^ homeostasis, activation of BKCa channels, and proteasome inhibition. BKCa: Ca^2+^‐activated K^+^; ER: endoplasmic reticulum; IGFIR: insulin‐like growth factor‐I receptor; MAPK: MAP kinase; ROS: reactive oxygen species.

The induction of paraptosis has evolved through the last two decades, and currently, involves several key mechanisms (Fontana et al., 2020; Hanson et al., 2023; Hoa et al., 2009):

1. Ion Channel Activation: Paraptosis can be initiated through the activation of large potassium (BK) channels, which are triggered by reactive oxygen species (ROS). This ion channel activation leads to cellular swelling and vacuolization.
2. ROS overproduction/ATP depletion: The activation of BK channels and subsequent cellular processes lead to a depletion of intracellular ATP, which is crucial for cell survival. Being the storehouse of ATP production, mitochondria, is also the site for ROS production due to oxidative damage; therefore, a decrease in the mitochondrial membrane potential results in increased ROS generation.
3. ER stress‐mediated paraptosis: Paraptosis being a PCD, requires new protein synthesis. The inhibition of paraptosis when treated with cycloheximide/actinomycin D suggested that translation and transcription are necessary for paraptosis induction. The requirement for protein synthesis brings our attention to the ER which functions as the mainstay for protein synthesis and sorting. Several ER‐localized proteins help to render this function. Studies have reported that the accumulation of newly synthesized misfolded proteins in the ER leads to ER stress and unfolded protein response which can be due to proteasomal inhibition.
4. Ca^2+^ influx: Ca was demonstrated to be liberated from its warehouses within ER through specific channels like IP3 receptors to the mitochondria‐associated ER membranes until reaching mitochondria. The cellular Ca^2+^ overload or redistribution of Ca^2+^ is associated with the commencement of paraptosis.
5. Insulin‐like growth factors: Paraptosis is initiated by the activation of the Insulin growth factor 1 receptor (IGF‐IR) and its intracytoplasmic domain (IGFIR‐IC). This process is dependent on the receptor's kinase domain and the presence of its ligand. Further research revealed that the mitogen‐activated protein kinase (MAPK) pathways, specifically MAPK/ERK and JNK are activated during paraptosis. The study also found that ERK‐2's phosphorylation of caspase‐9 at Thr125 diverts the cell from apoptosis to paraptosis, a pathway that does not involve the cleavage of caspase‐9 by its zymogens. Additionally, the overexpression of AIP1/Alix was shown to inhibit MAPK phosphorylation and cell death, suggesting its role as an endogenous inhibitor of paraptosis (Figure 1).

## 4 NATURAL PRODUCTS‐INDUCED PARAPTOSIS

Natural products have been increasingly recognized for their potential as inducers of non‐canonical cell death pathways, including paraptosis, which is crucial in the fight against cancer. These substances, derived from various natural sources, are capable of initiating cell death mechanisms that differ from the traditional apoptotic pathway. The significance of natural products lies in their ability to overcome the resistance of cancer cells to apoptosis, offering a promising alternative to cancer therapy. For instance, certain compounds have been identified to promote paraptosis by affecting key cellular structures like the ER and mitochondria, leading to cell death (Chen et al., 2023; Greco et al., 2021). Several natural products have been proven to trigger cancer cell death via paraptosis and/or apoptosis. These bioactive secondary metabolites from different origins, a variety of chemical classes and with unique mechanism of action were listed in Table 1. The majority of these bioactive metabolites are low‐molecular weight organic molecules.

**TABLE 1:** Examples of secondary metabolites capable of triggering paraptosis in cancer cells.

Natural product	Chemical structure	Class	Mechanism	Ref.
6‐Shogaol	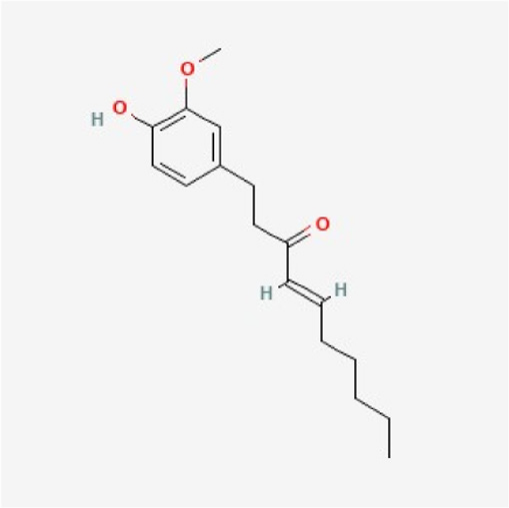	Phenol	Caspase‐independent paraptosis in cancer cells via proteasomal inhibition	Nedungadi et al. (2018)
Morusin	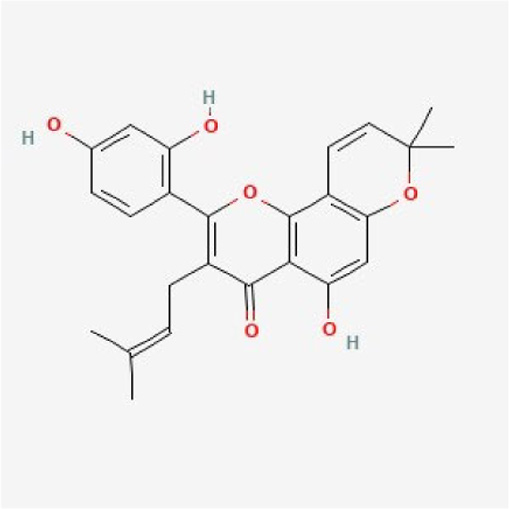	Flavonoid	Mitochondrial calcium overload and dysfunction, led to increased ROS levels and decreased mitochondrial membrane potential	Xue et al. (2018)
Curcumin	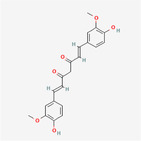	Phenol	Curcumin alters the expression of ER stress response genes and ER‐related miRNAs which participate in Akt‐Insulin and p53‐BCL2	Garrido‐Armas et al. (2018)
Cyclosporine A	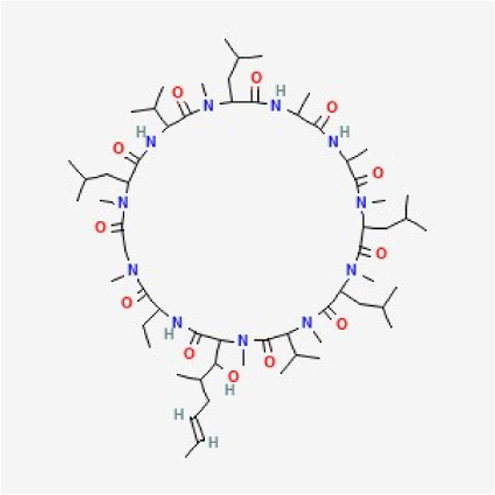	Cyclic peptide	AIP‐1/Alix downregulation, ER stress inhibition	Ram and Ramakrishna (2014)
Plumbagin	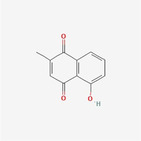	Phenol	Disruption of sulfhydryl homeostasis, Proteasome and ER stress inhibition	Binoy et al. (2019)
Jolkinolide B	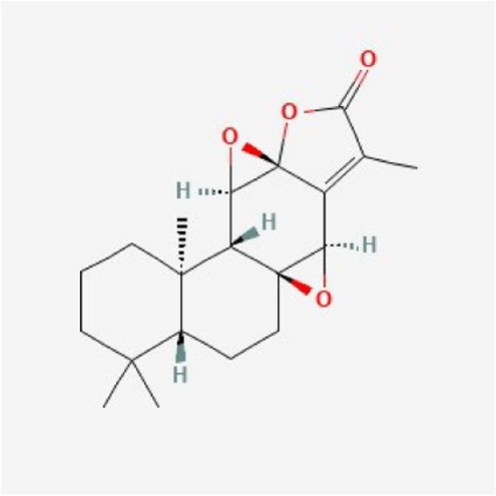	Diterpene	Trigger ROS‐mediated paraptosis to block the cancer growth of DDP‐resistant bladder cancer by targeting thioredoxin and glutathione systems	Sang et al. (2021)
Elaiophylin	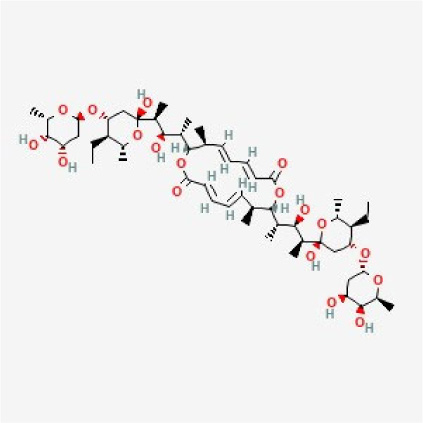	Macrodiolide	Overcome taxane, and PARPi resistance in ovarian cancer by regulating the SHP2/SOS1/MAPK‐mediated paraptosis pathway	Li et al. (2022)
Cannabidiol	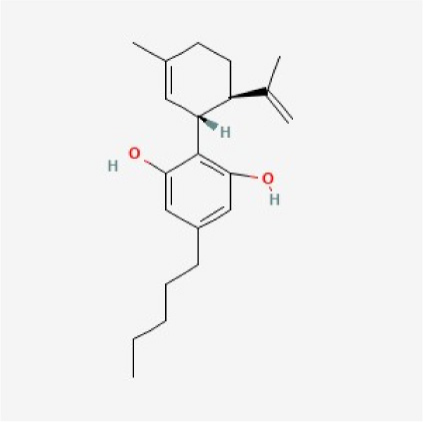	Polyketide	Upregulate ATF4 (activating transcription factor 4) and CHOP (CCAAT/enhancer‐binding protein homologous protein), elevate ER stress, and enhance ROS levels	Kim et al. (2024)
Glabridin	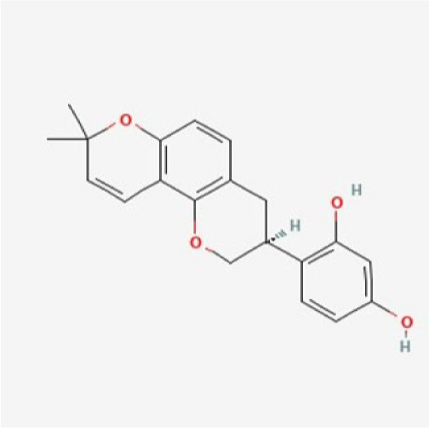	Flavonoid	ER stress, accumulation of poylubiquitinated proteins, matrix metalloprotinease loss	Cui and Cui (2022)
Epimedokoreanin B	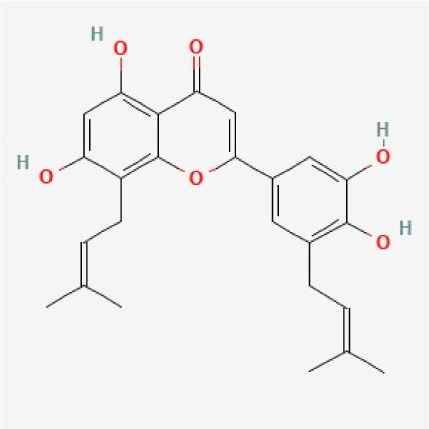	Flavonoid	Cytoplasmic vacuolation induction, downregulation of Alix	Zheng et al. (2022)

*Note*: All chemical structures were downloaded from PubChem repository.

Certain natural substances, including curcumin, morusin, and ophiobolin A, have been found to trigger paraptosis by disrupting ion balance within cells. Specifically, curcumin leads to paraptosis in ovarian cancer cells by causing an excess of calcium ions in mitochondria, leading to their enlargement and malfunction (Yoon et al., 2012). Morusin, a type of prenylflavonoid, similarly induces paraptosis in breast cancer through mitochondrial calcium ion overload (Xue et al., 2018). In the case of glioblastoma, ophiobolin A causes paraptosis by upsetting potassium ion balance, mainly due to its inhibition of BKCa channels (Bury et al., 2013).

Not only small organic molecules, iturin A‐like lipopeptides produced by *Bacillus subtilis* initiated both apoptosis and paraptosis in heterogeneous human epithelial colorectal adenocarcinoma (Caco‐2) cells. These lipopeptides exerted their action through occurrence of many cytoplasmic vacuoles accompanied by ER dilatation and mitochondrial swelling and dysfunction. ER stress was accompanied by high Ca^2+^ as well as ROS. Besides, Caco‐2 apoptosis was concomitated with the upregulation of bax and bad genes and the downregulation of bcl‐2 (Zhao et al., 2019).

Paraptosis induction, due to its distinct molecular action, aids in boosting the performance of proteasome inhibitors against cancer cells. Despite the antitumor potential of many proteasome inhibitors, their real‐world effectiveness often falls short due to inherent and acquired resistance. Thus, using a combination treatment approach is a promising way to overcome this resistance. For example, when initiating paraptotic cell death, the drug bortezomib, which inhibits the 20S core particle of the proteasome, can be used alongside loperamide, a medication typically used to treat diarrhea, to increase bortezomib's efficacy and minimize adverse effects, offering a potent treatment against colon cancer (Kim et al., 2019).

Paraptosis, a PCD pathway leading to cellular necrosis, has been also shown to enhance tumor immunogenicity. Prolonged activation of paraptosis can improve tumor immunogenicity, replicating the vaccinating effects of certain treatments. This process has been highlighted as a valuable strategy for clinical immunotherapy against cancer (Wepsic & Hoa, 2023). Recent findings suggest that paraptosis induces antitumor T‐cell immunity and other immunogenic responses against cancer, making it a crucial cell death mechanism in generating an antitumor effect in various cancer subtypes (Hanson et al., 2023). Studies have revealed that targeting specific molecules like valosin‐containing protein (VCP) can induce paraptosis in cancer cells, offering a potential avenue for novel therapeutic approaches (Lee et al., 2024). Additionally, research has shown that ablation of the ER stress kinase PERK can induce paraptosis and type I interferon, promoting antitumor T‐cell responses (Mandula et al., 2022). These studies collectively demonstrate the role of paraptosis in triggering immune responses against cancer cells, highlighting its potential in cancer therapy.

A few clinical trials are testing the efficacy and safety of paraptosis in cancer treatment. For example, the study discussed osimertinib, an epidermal growth factor receptor tyrosine kinase inhibitor (EGFR‐TKI), which has been shown to induce paraptosis in glioblastoma cells. A clinical trial using osimertinib on glioblastoma is currently ongoing (NCATS 1‐UH2‐TR001370‐01) (Hu et al., 2023). Additionally, although not explicitly stated as a clinical trial, the discovery of elaiophylin's ability to trigger paraptosis and overcome drug‐resistant tumors in ovarian cancer provides a strong foundation for developing new therapeutic strategies based on paraptosis induction (Li et al., 2022). These examples demonstrate the growing interest in exploring paraptosis as a viable approach to cancer therapy via using the safer, nature‐derived compounds.

The use of natural products in cancer treatment presents several challenges that hinder their effective application in clinical settings. One major issue is the lack of rigorous clinical trials to establish safety and efficacy, as many dietary supplements and natural compounds have not undergone the necessary FDA review for cancer therapy approval. This creates uncertainty for oncologists advising patients on which supplements may be beneficial or harmful, especially since nearly half of cancer patients report using new dietary supplements after diagnosis (Paller et al., 2016). Furthermore, the development of natural products into drugs is complicated by difficulties in large‐scale isolation, understanding their mechanisms of action, and ensuring consistent quality and potency across batches, as natural products often vary in composition due to environmental factors (Huang et al., 2021). The high costs associated with conducting comprehensive clinical trials, coupled with the low patentability of many natural compounds, further deter pharmaceutical companies from investing in natural product research. Additionally, there are challenges in identifying suitable models to evaluate the anticancer potential of these compounds, as cancer heterogeneity means that a compound effective in one model may not work in another (Mali, 2023). These factors contribute to a significant gap between the discovery of promising natural compounds and their translation into effective cancer therapies.

## FUNDING INFORMATION

This study acquired no external funding.

## CONFLICT OF INTEREST STATEMENT

Author declares no known conflicts of interest.

## Data Availability

Not applicable.
